# Bullous Systemic Lupus Erythematosus Associated with Esophagitis Dissecans Superficialis

**DOI:** 10.1155/2015/930683

**Published:** 2015-03-02

**Authors:** Meera Yogarajah, Bhradeev Sivasambu, Eric A. Jaffe

**Affiliations:** Department of Medicine, Interfaith Medical Center, Brooklyn, NY 11213, USA

## Abstract

Bullous systemic lupus erythematosus is one of the rare autoantibody mediated skin manifestation of systemic lupus erythematosus (SLE) demonstrating subepidermal blistering with neutrophilic infiltrate histologically. We present a case of a 40-year-old Hispanic female who presented with a several months' history of multiple blistering pruritic skin lesions involving the face and trunk, a photosensitive rash over the face and neck, swelling of the right neck lymph node, and joint pain involving her elbows and wrist. Her malady was diagnosed as bullous systemic lupus erythematosus based on the immunological workup and biopsy of her skin lesions. The patient also complained of odynophagia and endoscopy revealed esophagitis dissecans superficialis which is a rare endoscopic finding characterized by sloughing of the esophageal mucosa. The bullous disorders typically associated with esophagitis dissecans superficialis are pemphigus and rarely bullous pemphigoid. However, this is the first reported case of bullous systemic lupus erythematosus associated with esophagitis dissecans superficialis.

## 1. Introduction

Bullous systemic lupus erythematosus is a subepidermal blistering disease mediated by autoantibodies that occurs in patients with SLE. The first case of bullous systemic lupus erythematosus was described in 1973. Esophagitis dissecans superficialis is a rare endoscopic finding characterized by sloughing of the entire length of the esophageal mucosal epithelium. Esophagitis dissecans superficialis has not been described with bullous systemic lupus erythematosus.

## 2. Case Report

A 40-year-old Hispanic woman with bronchial asthma and major depression was admitted complaining of a several months' history of multiple blistering pruritic skin lesions involving her face and trunk, a photosensitivity rash over her face and neck, swelling of a lymph node in her right neck, and joint pain involving her elbows and wrists. These symptoms were associated with hair loss. She also complained of painful swallowing of both solids and liquids. However, she denied symptoms of Raynaud's phenomenon, exertional dyspnea, hematuria, and proximal muscle weakness or pain.

On examination, she had pink conjunctiva, an erythematous malar rash, painless superficial oral ulcers, and a solitary nontender right-sided cervical lymph node. She also had vesicular lesions over her face and trunk with excoriations. Her elbows and wrist joints were tender but without swelling. Examination of her central nervous system was normal.

Our differential diagnosis included bullous systemic lupus erythematosus, epidermolysis bullosa acquisita, dermatitis herpetiformis, and bullous pemphigoid.

Initial workup revealed a normal complete blood count and comprehensive metabolic panel. Her autoimmune workup was significantly positive for anti-nuclear antibody (ANA) with a titer of 279 IU/mL (NR < 7.5), anti-double-stranded DNA antibody (ds-DNA) with a titer of 119 IU/mL (NR < 9), and anti-Smith antibody with a titer >8 AI (NR < 0.9). Tests for anti-ribonucleoprotein (anti-RNP) antibody, anti-centromere antibody, anti-topoisomerase-1 (anti-SCL-70), anti-Jo-1 antibody, anti-SSA antibody, and anti-SSB antibody were negative. Her erythrocyte sedimentation rate (ESR) was 18 and C-reactive protein (CRP) was 1.8 mg/L (normal 0–4.8) and complement levels were normal. Anti-neutrophil cytoplasmic antibody (ANCA) panel was negative. Biopsy of her cervical lymph showed follicular hyperplasia.

**Figure 1:**
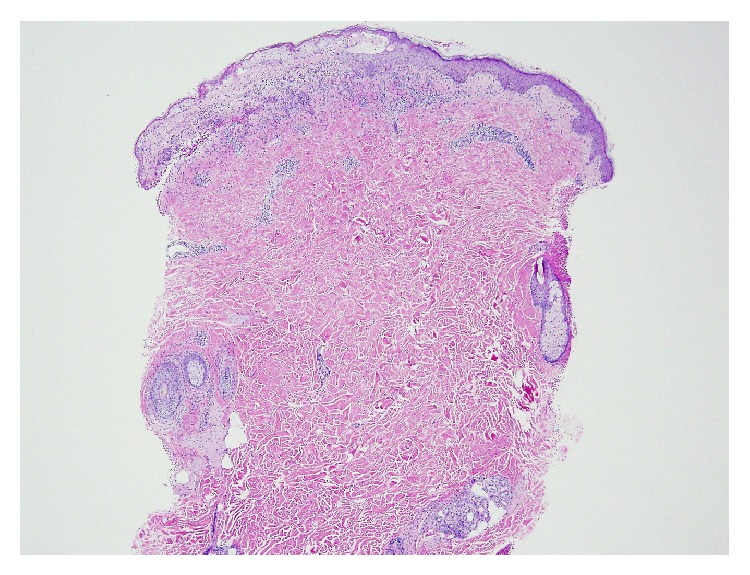
A low power (4x) skin H&E image reveals superficial band-like and perivascular inflammatory infiltrate with overlying epidermal necrosis and vesiculation.

**Figure 2:**
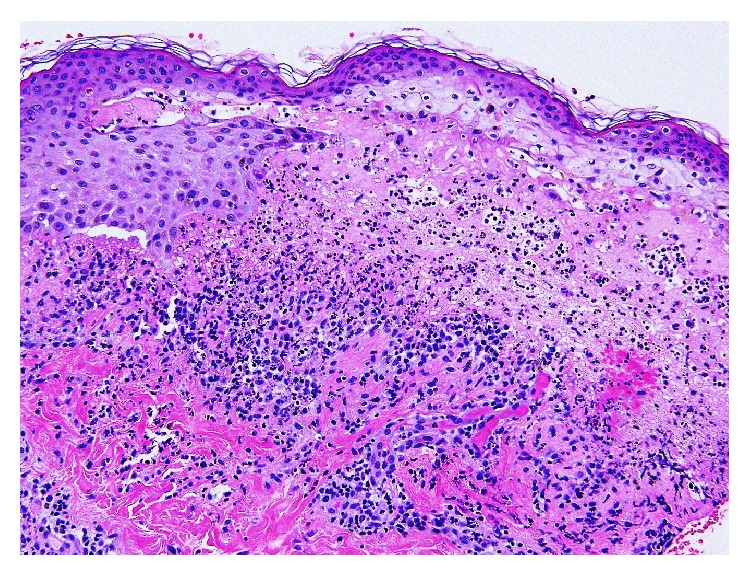
A higher power (20x) skin H&E image reveals abundant neutrophils and scattered eosinophils with a focal subepidermal split. Epidermal necrosis is again noted.

Skin biopsy showed subepidermal vesicular dermatitis with overlying epidermal necrosis, predominantly neutrophilic infiltrate and focal interface changes (Figures 1 and 2). These findings suggested the presence of an immunoblistering process or a bullous drug eruption. Direct immunofluorescence did not show any abnormal deposits of IgG, IgM, IgA, C3, or fibrin in the epidermis or blood vessels but did reveal linear deposits of C3 and IgG and weak linear deposit of IgM in the basement membrane. No IgA or fibrin deposits were detected in the basement membrane.

These findings can be seen in bullous pemphigoid, epidermolysis bullosa acquisita, and bullous systemic lupus erythematosus. A direct immunofluorescence of the salt-split skin demonstrated linear deposit of C3 and IgG and weak linear deposit of IgM on the dermal floor side of the salt-split skin which was consistent with bullous systemic lupus erythematosus as the patient also fulfilled the ACR criteria for diagnosis of SLE.

However, as the patient also complained of painful swallowing, mixed connective tissue disease with dysphagia due to systemic sclerosis was a concern. Tests for anti-ribonucleoprotein (anti-RNP) antibody, anti-centromere antibody, anti-topoisomerase I (anti-SCL70), and anti-Jo-1 antibody were negative. Polymyositis was unlikely as patient did not have any muscle weakness and her creatine kinase and aldolase levels were normal.

**Figure 3:**
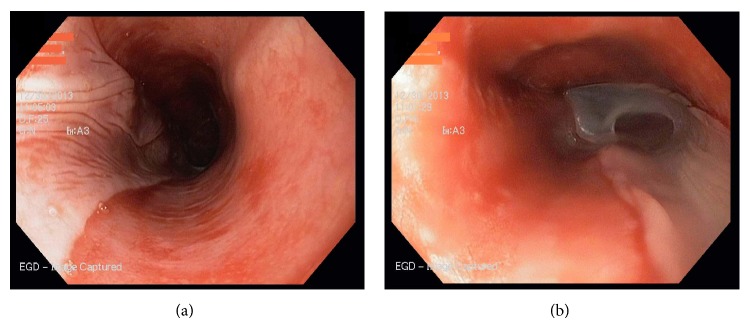
EGD showing vertical fissures in the distal esophagus with sloughing of the mucosa.

She underwent endoscopy which showed sloughing of the esophageal mucosa suggestive of esophagitis dissecans superficialis (Figures 3(a) and 3(b)) and biopsy revealed chronic inflammatory changes with no evidence of yeast or pseudohyphae invasion.

We are presenting a case of bullous systemic lupus erythematosus associated with esophagitis dissecans superficialis. There have been reported cases of esophagitis dissecans superficialis associated with bullous autoimmune disorders mainly pemphigus. However, this is the first reported case of esophagitis dissecans superficialis associated with bullous systemic lupus erythematosus.

## 3. Discussion

Bullous systemic lupus erythematosus is an autoantibody mediated subepidermal blistering disease that occurs in patients with SLE [1]. The first case of bullous systemic lupus erythematosus was described by Pedro and Dahl in 1973 [2].

Severe cutaneous lupus erythematosus can present as vesiculobullous lesions due to extensive damage of epidermal basal layer. As all bullous lesions occurring in SLE patients are not bullous systemic lupus erythematosus, a criterion to diagnose bullous systemic lupus erythematosus was initially proposed by Camisa and Sharma [3]. Then Gammon and Briggaman [4] classified bullous systemic lupus erythematosus into two subtypes, type 1 with circulating antibodies and type 2 without circulating antibodies. Currently, bullous systemic lupus erythematosus is classified into 3 subtypes [5]. The diagnosis of bullous systemic lupus erythematosus type 1 requires all five of the following. But types 2 and 3 could be diagnosed with the first four criteria [5, 6]:

- ACR criteria for the diagnosis of SLE,
- acquired vesiculobullous eruption,
- histologic evidence of a subepidermal blister and a predominantly neutrophilic dermal infiltrate,
- direct immunofluorescence (DIF) microscopy demonstrating IgG with or without IgA and IgM deposits at the basement membrane zone (BMZ),
- evidence of antibodies to type VII collagen via direct immunofluorescence (DIF) microscopy or indirect immunofluorescence (IIF) on salt-split skin, immunoblotting, immunoprecipitation, enzyme-linked immunosorbent assay (ELISA), or immunoelectron microscopy.

All of the above criteria were fulfilled in our patient and allowed us to make the diagnosis of bullous systemic lupus erythematosus type 1.

The differential diagnosis of blistering eruptions in patients with SLE includes dermatitis herpetiformis [5], bullous pemphigoid [7, 8], epidermolysis bullosa acquisita [9], and bullous systemic lupus erythematosus [10, 11]. Dermatitis herpetiformis [12] can be differentiated by light microscopy and direct immunofluorescence. Bullous pemphigoid, epidermolysis bullosa acquisita, and bullous systemic lupus erythematosus demonstrate similar direct immunofluorescence patterns. Salt-split skin test [13] demonstrates IgG antibodies on the epidermal side (roof pattern) of the split skin in bullous pemphigoid as opposed to epidermolysis bullosa acquisita [14] and bullous systemic lupus erythematosus [15] that demonstrate IgG antibodies on the dermal side (floor pattern) of the split skin and therefore is difficult to differentiate. Presence of ACR criteria for the diagnosis of SLE favors the diagnosis of bullous systemic lupus erythematosus.

Esophagitis dissecans superficialis is a rare endoscopic finding characterized by sloughing of the entire length of the esophageal mucosal epithelium. This was first described by Carmack [16]. It has been associated with bisphosphonates [17], nonsteroidal anti-inflammatory drugs [15], celiac disease [18], collagen disease [19], and autoimmune bullous disease typically pemphigus vulgaris [20]. There have been few reported cases associated with bullous pemphigoid [21]. However, there have been no reported cases of bullous systemic lupus erythematosus associated with esophagitis dissecans superficialis. It is very rare to get esophagitis dissecans superficialis with a subepidermal blistering disorder as it has antibodies against the epithelial basement membrane as in bullous systemic lupus erythematosus and the treatment of choice is steroids. Our patient was started on steroids and the odynophagia improved.

In conclusion, bullous skin lesions can be seen in SLE and though is a rare skin manifestation, warrants a skin biopsy with immunofluorescence studies to make a diagnosis of bullous systemic lupus erythematosus. The treatment of choice for bullous systemic lupus erythematosus is dapsone [22]. Steroid is the other alternative drug for patients who fail to respond to dapsone, have intolerance to dapsone, or have other systemic manifestations of SLE warranting steroid treatment. Other therapeutic options include methotrexate (MTX), azathioprine, mycophenolate mofetil, and rituximab.

This case highlights the importance of endoscopy in a patient with bullous systemic lupus erythematosus who complains of odynophagia as it is necessary to make the correct diagnosis since esophagitis dissecans superficialis and bullous systemic lupus erythematosus are treated differently.
